# Improved cardiac motion self-gating

**DOI:** 10.1186/1532-429X-15-S1-P83

**Published:** 2013-01-30

**Authors:** Fei Han, Stanislas Rapacchi, Peng Hu

**Affiliations:** 1 Radiology, UCLA, Los Angeles, CA, USA

## Background

Cardiac motion self-gating is a technique where MRI signal is used to derive motion triggers instead of ECG, which might be problematic in high B0 field or cases where ECG is not accessible (e.g. fetal cardiac imaging). However, the performance of existing cardiac self-gating approaches have not yet enabled clinical utility. We propose and evaluate here a novel cardiac self-gating strategy that potentially improves the trigger detection accuracy and reliability.

## Methods

**Figure 1:**
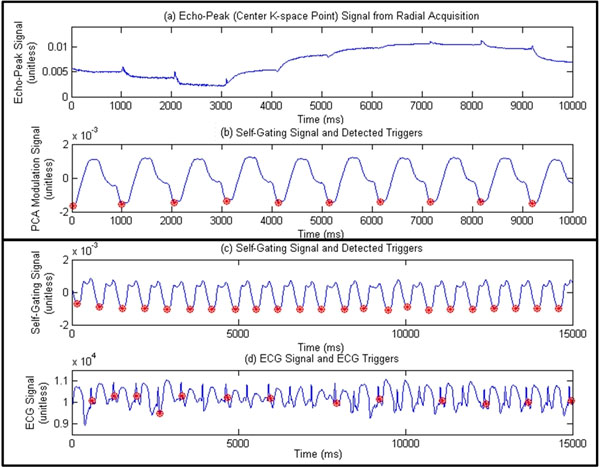
a) Center k-space point from radial acquisition, which was used in conventional cardiac self-gating method, shows cardiac motion signal with severe drifting and distortion; b) self-gating signal and triggers (marked by '*') detected by the proposed method on the same subject; c) self-gating and d) ECG signal with triggers on a different subject using a 3T scanner where the ECG fails to provide accurate triggers while the proposed method offers stable triggers.

Conventional cardiac self-gating uses the k-space center from a radial acquisition to represent the cardiac motion and derive triggers. However, this strategy suffers from signal drifting and distortion shown in Fig.1a. We hypothesize that this is because the k-space center signal was modulated by the eddy currents from the varying phase-encoding(PE) or radial acquisition gradients. Such interference should be removed for robust self-gating. To test our hypothesis, we ran a Cartesian breath-held cardiac cine sequence with phase-encoding gradient turned off. We used Principle Component Analysis(PCA) to extract the cardiac motion signal from the acquired data. Trigger is then detected by finding the local maximum with an adaptive threshold. Our method differs from other cardiac self-gating techniques in four aspects: 1)The whole k-space center-line is used instead of the center point only; 2)Coil arrays were used instead of a single coil; 3)The self-gating signal is derived from repeatedly acquired non-phase-encoded k-space center-line and is therefore free of aforementioned signal interference. 4)PCA is used to further reduce any residual interference.

**Figure 2:**
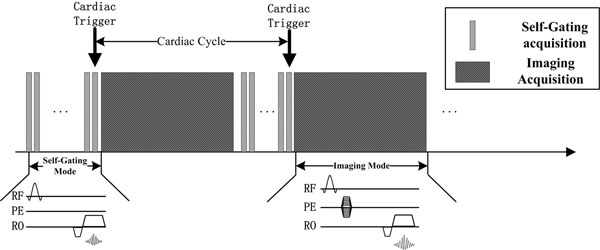
Implementation of the proposed cardiac self-gating method on a cardiac imaging sequence, which switches between self-gating mode and imaging mode. Our results support our hypothesis that such a strategy will enable higher accuracy and reliability in cardiac motion self-gating. Current development is ongoing to implement this sequence and evaluate the accuracy of self-gating using this approach on healthy subjects.

Fig.2 shows a potential implementation in a cardiac MRI sequence. It consists of a self-gating mode where the k-space center-line is repeatedly acquired and an imaging mode where k-space is sampled. The sequence switches from self-gating mode, where the PE gradients are turned off, to imaging mode when a new trigger is detected and switches back after imaging to wait for the next trigger.

## Results

Fig.1b shows the cardiac self-gating signal and trigger generated by the proposed method on the same subject for Fig.1a. Fig.1c&d show the result from a 3T scanner where the quality of ECG is poor while our self-gating method could still provide accurate triggers. Based on data from 8 healthy volunteers, the overall trigger detection rate was 99% (one failed due to non-ideal breath-holding) and the average temporal variability of triggers was ±7.79ms using the ECG as reference. On 3 subjects using the k-space center point only as previously described, the overall detection rate was only 65%.

## Conclusions

Our data demonstrates that the proposed cardiac self-gating method can significantly reduce the drift and distortion of the self-gating signal and therefore improve cardiac trigger detection accuracy and reliability. Future work will be focused on implementing the technique in an imaging sequence as Fig.2.

## Funding

The authors acknowledge research-funding support from AHA (10SDG4200076), NIH (1R21HL113427).

